# ^1^H, ^13^C, and ^15^N resonance assignments of the *C*-terminal lobe of the human HECT E3 ubiquitin ligase ITCH

**DOI:** 10.1007/s12104-018-9843-2

**Published:** 2018-09-18

**Authors:** Steven A. Beasley, Roela Bardhi, Donald E. Spratt

**Affiliations:** Gustaf H. Carlson School of Chemistry and Biochemistry, Clark University, 950 Main St., Worcester, MA 01610 USA

**Keywords:** ITCH, Atrophin-1-interacting protein 4, Ubiquitin, HECT, E3 ubiquitin ligase, Ubiquitylation, NMR spectroscopy

## Abstract

ITCH (*aka* Atrophin-1-interacting protein 4) is a prominent member of the NEDD4 HECT (*H*omologous to *E*6AP *C-T*erminus) E3 ubiquitin ligase family that regulates numerous cellular functions including inflammatory responses through T-cell activation, cell differentiation, and apoptosis. Known intracellular targets of ITCH-dependent ubiquitylation include receptor proteins, signaling molecules, and transcription factors. The HECT *C*-terminal lobe of ITCH contains the conserved catalytic cysteine required for the covalent attachment of ubiquitin onto a substrate and polyubiquitin chain assembly. We report here the complete experimentally determined ^1^H, ^13^C, and ^15^N backbone and sidechain resonance assignments for the HECT *C*-terminal lobe of ITCH (residues 784–903) using heteronuclear, multidimensional NMR spectroscopy. These resonance assignments will be used in future NMR-based studies to examine the role of dynamics and conformational flexibility in HECT-dependent ubiquitylation as well as deciphering the structural and biochemical basis for polyubiquitin chain synthesis and specificity by ITCH.

## Biological context

Ubiquitylation is an important posttranslational modification that maintains cellular health and homeostasis by targeting proteins for proteosomal or autophagic degradation (Cohen-Kaplan et al. 2016). Ubiquitylation occurs through the sequential transfer of ubiquitin between three enzymes—ubiquitin-activating enzyme (E1), ubiquitin-conjugating enzyme (E2), and ubiquitin ligase (E3)—ultimately resulting in the covalent attachment of ubiquitin on to a substrate via an isopeptide bond. In contrast to the RING (*R*eally *I*nteresting *N*ew *G*ene) E3 ubiquitin ligases that primarily act as scaffolds by orienting the E2 ~ ubiquitin thiolester complex and target protein for ubiquitin transfer, the HECT (*H*omologous to *E*6AP *C*-*T*erminus) E3 ubiquitin ligases play a catalytic role in the final attachment of ubiquitin by forming a thiolester intermediate with ubiquitin before transferring it to a substrate protein (Lorenz 2018; Metzger et al. 2012). The combinatorial effect of ~ 40 human E2 enzymes and hundreds of E3 ligases allows for the observed diversity of ubiquitylation substrate specificity.

In humans, there are 28 members of the HECT E3 ubiquitin ligase family (Scheffner and Kumar 2014) with each containing a conserved ~ 350 residue HECT domain near their *C*-termini. Each HECT domain is comprised of two lobes—a larger *N*-terminal lobe responsible for recruiting an E2 ~ ubiquitin complex, and a smaller *C*-terminal lobe that contains the catalytic cysteine required to covalently attach ubiquitin to its substrates (Lorenz 2018). The HECT E3 ubiquitin ligase family can be further categorized into subfamilies, which includes the most well-examined NEDD4 subfamily. The NEDD4 HECT E3 ubiquitin ligases are comprised of a Ca^2+^-binding C2 domain, WW domains that bind the PPxY protein–protein interaction motif, as well as the HECT domain required for its ubiquitylation activity. The 3D structures of several NEDD4 HECT domains family members have been solved by X-ray crystallography, either alone or complexed with E2 and/or ubiquitin (Lorenz 2018), however, there are many unanswered questions regarding how these enzymes assemble into multi-protein complexes to synthesize polyubiquitin chains.

ITCH, also known as Atrophin-1-interacting protein 4, is a prominent member of the NEDD4 subfamily that regulates signaling pathways involved in immune-cell differentiation, control of the inflammatory signaling pathways, and apoptosis (Aki et al. 2015). ITCH is an intriguing HECT E3 ubiquitin ligase due to its ability to synthesize different polyubiquitin lysine linkages (i.e. K29, K48, and/or K63-linked) depending on the substrate it modifies, and this ubiquitin linkage specificity is dependent on the last 60 amino acids of the *C-*terminal lobe of ITCH (Kim and Huibregtse 2009). For example, ITCH has been observed to build K29-linked polyubiquitin chains on the transmembrane receptor protein Notch that leads to Notch undergoing endocytosis and lysosomal degradation (Chastagner et al. 2008). ITCH knockout mice display an ‘Itchy’ phenotype caused by inflammatory dysregulation due to unfettered Notch signaling (Chastagner et al. 2008; Matesic et al. 2008). ITCH negatively regulates the Hippo tumor suppressor pathway by building a K48-linked polyubiquitin chain on of the serine/threonine kinase LATS1, a tumor growth inhibitor that induces G2-M arrest and apoptosis (Salah et al. 2014). ITCH also controls intracellular concentrations of p63 and p73, members of the tumor suppressor protein family, by K48-polyubiquitylation to target these oncoproteins for proteosomal degradation (Melino et al. 2008). K63-linked ITCH-dependent polyubiquitination has also been observed for the transcription factor p45/NF-E2 causing it to migrate out of the nucleus into the cytoplasm and thus inactivating p45/NF-E2 (Lee et al. 2008).

Presently there is no structural rationale for how ITCH is capable of building these different polyubiquitin chain types and the catalytic mechanism for ITCH is currently unknown. Here we report the complete backbone and sidechain ^1^H, ^13^C, and ^15^N resonance assignments for the catalytic *C*-terminal lobe of ITCH (residues 784–903) using 3D heteronuclear NMR spectroscopy. These resonance assignments will enable future structural and mechanistic studies to better understand ITCH-dependent ubiquitylation.

## Methods and experiments

### Protein expression and purification

The human HECT *C*-terminal lobe of ITCH (Uniprot: Q96J02, residues 784–903) with C835S and C855S substitutions was synthesized and codon-optimized by DNA2.0 (Newark, CA, USA) and cloned into an ampicillin resistant T7-inducible vector with an *N*-terminal polyhistidine tag (His_6_-tag) followed by a TEV protease cleavage site (ENLYFQ). The His_6_-TEV-ITCH *C*-terminal lobe construct was transformed into *E. coli* BL21(DE3) RIL+ and grown at 37 °C in M9 media (2 × 1 L) supplemented with ^15^NH_4_Cl (1 g/L), ^13^C_6_-glucose (2 g/L), 100 mg/L ampicillin and 34 mg/L chloramphenicol. When the culture reached an OD600 of 0.8, the cultures were induced with 1 mM IPTG at 16 °C for 20 h. The cells were harvested by centrifugation at 6000×*g* for 10 min using a Sorvall LYNX 4000 superspeed centrifuge with a Fiberlite F 10 − 4 × 1000 LEX Carbon Fiber rotor (Thermo-Fisher). Cell pellets were resuspended in 20 mL of wash buffer (50 mM Na_2_HPO_4_ pH 8.0, 300 mM NaCl, 10 mM imidazole) with 1 mM PMSF and an EDTA-free protease inhibitor mini tablet (Pierce), lysed using an Emulsiflex-C5 homogenizer (Avestin, Ottawa, ON, Canada), and clarified by ultracentrifugation using a Optima L-80 XP ultracentrifuge with a 70.1 Ti rotor (Beckman-Coulter) at 41,000 rpm for 40 min. The clarified supernatant was then applied to 5 mL of HisPur Ni–NTA resin (Thermo-Fisher) pre-equilibrated with wash buffer at a flow rate of 0.5 mL/min. After the resin was washed with 25 column volumes of wash buffer, the protein was eluted with 20 mL of elution buffer (50 mM Na_2_HPO_4_ pH 8.0, 300 mM NaCl, 250 mM imidazole). Fractions containing eluted protein were pooled and incubated with recombinant TEV protease for 1 h at 25 °C (1 mg TEV/50 mg eluted protein) to cleave the His_6_-tag, then dialyzed against wash buffer stirring overnight at 4 °C. The TEV cleaved protein was then reapplied to 5 mL of HisPur Ni–NTA resin at a flow rate of 0.5 mL/min and the flowthrough containing ^13^C–^15^N-labeled ITCH *C*-terminal lobe was collected and pooled. The protein was then concentrated using a Amicon 15 mL centrifugal filter with a 10 kDa MWCO (Millipore) and loaded onto a HiLoad 16/60 Superdex75 column equilibrated with 20 mM MES, 120 mM NaCl, 1 mM EDTA, 2 mM TCEP, pH 6.0 at a flow rate of 1 mL/min using an ÄKTA pure 25L FPLC (GE Healthcare Life Sciences). Fractions containing the purified ^13^C–^15^N-labeled ITCH *C*-terminal lobe, as assessed by SDS-PAGE, were pooled and concentrated. The resulting ITCH *C*-terminal lobe protein had an additional “GS” at its *N*-terminus as a result of its cloning and TEV cleavage. After purification, the concentration of the protein was determined using the Bradford assay (Bio-Rad) or a A_280_ using a Nanodrop One C UV/Vis spectrophotometer (Thermo-Fisher).

### NMR spectroscopy

The NMR samples used for resonance assignment of ^13^C–^15^N-labeled human ITCH *C*-terminal lobe were prepared in 20 mM MES, 120 mM NaCl, 1 mM EDTA, 2 mM TCEP, 10% D_2_O/90% H_2_O at pH 6.0. The samples were concentrated to a final volume of 600 μL and transferred to a 5 mm O.D. thin walled NMR tube (New-Era). Imidazole (1.6 mM) was added as an internal pH indicator to monitor the pH of the sample during data acquisition (Baryshnikova et al. 2008).

All NMR data were collected at 25 °C using a Varian Inova 600 MHz 4-channel solution-state NMR Spectrometer equipped with a 5-mm PFG triple-resonance probe housed and maintained in the Sackler Sciences Center at Clark University. Backbone assignments and aliphatic side chain assignments were determined using the following standard pulse sequences in the Varian Biopack in VnmrJ 3.0: ^1^H–^15^N HSQC, aliphatic ^1^H–^13^C HSQC, HNCO, HN(CA)CO, HNCA, HN(CO)CA, HNCACB, and CBCA(CO)NH, aliphatic HCCH-TOCSY, C(CO)NH, H(CCO)NH, and ^1^H–^15^N NOESY. Aromatic sidechain assignments were determined using an aromatic ^13^C-HSQC, HBCBCGCDHD and HBCBCGCDCEHE experiments in combination with an aromatic HCCH-TOCSY and aromatic ^13^C-NOESY experiments. All data were processed using NMRPipe and NMRDraw (Delaglio et al. 1995) and analyzed using NMRViewJ (Johnson and Blevins 1994). All of the relevant peak lists and the complete ^1^H, ^13^C, and ^15^N backbone and sidechain chemical shift assignments have been deposited into the Biological Magnetic Resonance Databank (http://www.bmrb.wisc.edu) under ascension code 27477.

### Assignments and data deposition

**Fig. 1:**
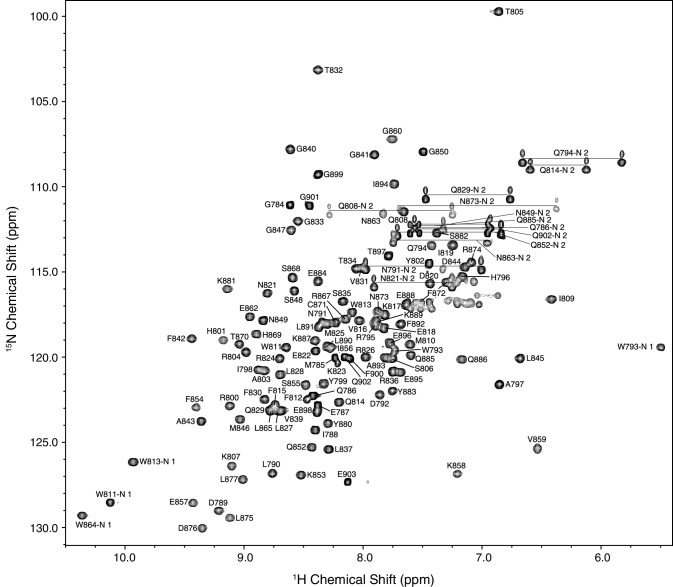
Assigned observable ^1^H–^15^N HSQC spectrum of the human HECT *C*-terminal lobe of ITCH (residues 784–903, 2.6 mM). The spectrum is labeled according to the one-amino acid code and residue number of the human ITCH sequence. The NMR sample contained ^13^C and ^15^N-isotopically enriched ITCH in 20 mM MES pH 6.0, 120 mM NaCl, 1 mM EDTA, and 10% D_2_O. Data was collected on a Varian Inova 600-MHz NMR spectrometer at 25 °C. Peaks corresponding to asparagine and glutamine side chain amides are connected with a horizontal line

The non-artifact and non-proline amide protons (113/115; 98.26%), backbone atoms (350/360; 97.22%) and ^1^H chemical shift assignments of the Hα (119/120, 99.17%) and Hβ (201/203, 99.01%) were definitively assigned. Every Cα and Cβ resonance were assigned except for the *N*-terminal artifact glycine and P878 (120/122, 98.36%). Aliphatic sidechain resonances of Cγ (60/62, 96.77%), Cδ (29/30, 96.67%), and Cε (13/13, 100%) were also assigned. Almost all nitrogen atoms—excluding the lysine, arginine, and histidine sidechain and proline backbone nitrogen atoms—were definitively assigned (130/132; 98.48%). The missing amide peaks for K861 and W864 could not be assigned possibly due to fast amide exchange with the solvent and/or high flexibility of the region. There were no unassigned peaks visible on the well-dispersed ^1^H–^15^N HSQC spectrum (Fig. 1). One residue of particular interest that will be followed in future chemical shift perturbation experiments is the catalytic cysteine of ITCH (C871) as it has distinct Cβ peaks on the ^1^H–^13^C HSQC whose chemical shift indicates that it is reduced (Kornhaber et al. 2006) as would be required for its ubiquitylation activity.

**Fig. 2:**
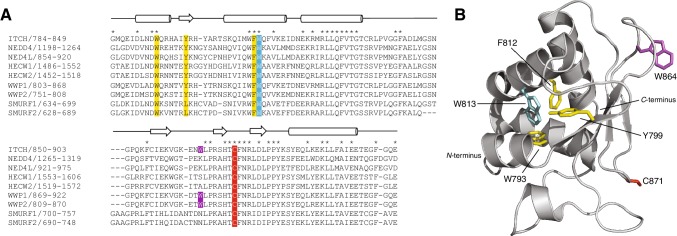
**a** Multiple sequence alignment of the *C*-terminal lobes of the NEDD4 HECT E3 ubiquitin ligase subfamily. The sequence alignment was performed using T-Coffee (Notredame et al. 2000) followed by manual curation in Jalview (Waterhouse et al. 2009). The α-helices and β-sheets based on the crystal structure of ITCH are shown as cylinders and arrows, respectively. Absolutely conserved residues are marked with an asterisk. **b** Structure of ITCH C-terminal lobe (PDB:3TUG) highlighting the catalytic cysteine C871 (red), as well as the W813 (cyan), surrounded by aromatic residues W793, Y799, and F812 (yellow). The solvent exposed tryptophan W864 is shown in magenta. Noteworthy residues discussed in the text are also highlighted in panel A using the same color scheme

**Fig. 3:**
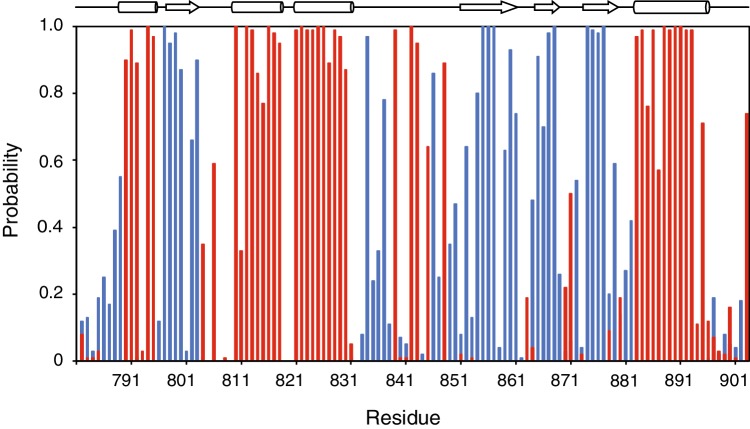
Predicted secondary structural regions of the ITCH *C*-terminal lobe. The probability plot was made by inputting the experimentally determined resonance assignments for ITCH into the online webserver CSI 3.0 (Hafsa et al. 2015). The propensity to form an a-helix or b-sheet are denoted in red and blue, respectively

ITCH is a member of the NEDD4 subfamily of HECT E3 ubiquitin ligases that shows a high degree of sequence conservation (Fig. 2a) with many of these conserved residues found within the hydrophobic core of the protein. For example, there are exceptional upfield peaks of the W813 aromatic side chain in both the ^1^H–^15^N HSQC and aromatic ^1^H–^13^C HSQC, likely due to shielding effect within the hydrophobic core surrounded by three proximal aromatic residues (i.e. W793, Y799, and F812; Fig. 2b). Intriguingly, there is one exceptional tryptophan (W864) on the surface that is found only in ITCH, as well as WWP1 (W883) and WWP2 (W823). This residue, which resides on the same face of the protein as the catalytic cysteine, deserves further attention as the mechanism of ubiquitin chain building and lysine-linkage specificity depends on these subtle differences between the different NEDD4 subfamily members. The secondary structure based on the chemical shift index (CSI) 3.0 web server analysis of chemical shifts (Hafsa et al. 2015) is in good agreement with the known PDB structures of ITCH (Zhang et al. 2016) and other HECT domain *C*-terminal lobes with an αβα_2_β_3_α organization indicating that the protein is well folded and in the correct structural conformation (Fig. 3).

In conclusion, we present the complete backbone and side chain resonance assignments of the catalytic HECT *C-*terminal lobe of ITCH. These resonance assignments will be used to examine the role of inherent conformational flexibility within the *C*-terminal lobe of ITCH that will help us decipher how ITCH is capable of building K29, K48, and K63-linked polyubiquitin chains on its diverse intracellular substrates.

## Abbreviations

HECT: Homologous to E6AP *C*-terminus
NMR: Nuclear magnetic resonance
